# Relationship Between Cognitive Performance and Anthropometric Status, Dietary Diversity, and Diet Quality in Older Turkish Adults: A Cross-Sectional Study

**DOI:** 10.3390/jcm15187280

**Published:** 2026-09-19

**Authors:** Fatmanur Hümeyra Zengin, Tuğba Tatar, İrem Dursun, Reyhan Dalkıran, Gözde Ede İleri, Rüveyda Esra Özkalaycı

**Affiliations:** 1 Department of Nutrition and Dietetics, Faculty of Health Sciences, Kastamonu University, 37150 Kastamonu, Türkiye; fhzengin@kastamonu.edu.tr (F.H.Z.); ttatar@kastamonu.edu.tr (T.T.); irremdrsn@gmail.com (İ.D.); reyhandalkiran61@gmail.com (R.D.); 2 Department of Nutrition and Dietetics, Faculty of Health Sciences, Çankırı Karatekin University, 18100 Çankırı, Türkiye; gozdeede@karatekin.edu.tr; 3 Department of Nutrition and Dietetics, Faculty of Health Sciences, Recep Tayyip Erdoğan University, 53100 Rize, Türkiye

**Keywords:** body mass index, cognitive performance, dietary diversity, diet quality, older adults, Montreal Cognitive Assessment

## Abstract

**Background/Objectives:** The increasing prevalence of mild cognitive impairment and dementia among older adults represents a major public health concern. This study aimed to examine the relationships between anthropometric status, dietary diversity, dietary quality, and cognitive performance in older adults. **Methods:** This study was a cross-sectional study. Anthropometric measurements were performed. The Dietary Diversity Score (DDS), Healthy Eating Index-2020 (HEI-2020), 24 h dietary recall and the Montreal Cognitive Assessment (MoCA) were used. We used hierarchical multiple linear regression analysis. **Results:** Among 117 older adults, 53.0% screened positive for possible mild cognitive impairment (MCI) based on the MoCA. Participants screening positive for possible MCI had a higher body mass index (BMI) compared to those with normal cognitive performance (32.67 ± 5.78 and 29.54 ± 3.39 kg/m^2^; *p* < 0.001) and a higher prevalence of obesity (62.9% and 41.8%; *p* = 0.027). The final regression model explained 26.0% of the variance in MoCA scores (R^2^ = 0.260; adjusted R^2^ = 0.212; *p* < 0.001). More years of education were associated with higher MoCA scores (B = 0.768, β = 0.363, *p* < 0.001), while higher BMI was associated with lower MoCA scores (B = −0.301, β = −0.240, *p* = 0.010). **Conclusions:** In older adults, higher BMI values were found to be associated with lower MoCA scores in the adjusted model. In this sample, no significant correlation was found between DDS and HEI-2020 scores obtained from a single 24 h food intake record and MoCA scores. Longitudinal studies including repeated nutritional assessments are needed to clarify the direction of these relationships.

## 1. Introduction

According to United Nations data, the global population is rapidly aging. Individuals aged 65 years and older accounted for approximately 10% of the world’s population in 2022, and by 2100, they are expected to comprise nearly one-quarter of the global population [1]. In the context of this rapidly aging population, maintaining cognitive function and preventing age-related cognitive decline have become critical public health priorities. Recent years have highlighted that pharmacological interventions aimed at improving quality of life or preserving cognition in individuals with normal cognitive function, mild cognitive impairment (MCI), or dementia lack strong evidence, whereas the importance of non-pharmacological interventions and multidisciplinary care has been emphasized [2,3]. Non-pharmacological strategies such as dietary habits may influence biological aging and are associated with age-related conditions, including diabetes, cardiovascular disease, and dementia [4]. Identifying modifiable nutritional and anthropometric factors related to cognitive performance is important in clinical settings because both are routinely measured in outpatient care and can be targeted for intervention.

Healthy dietary patterns have been shown to support brain health and potentially reduce the risk of cognitive decline [5,6]. Observational studies have reported that higher intake of nutrients such as vitamins, minerals, carotenoids, fatty acids, and fiber may be associated with a reduced or increased risk of cognitive impairment and/or decline [7,8]. The findings from the Finnish Geriatric Intervention Study to Prevent Cognitive Impairment and Disability (FINGER) showed that higher adherence to healthy dietary patterns in older individuals was associated with better overall cognitive performance, and that improvements in diet quality may be linked to positive changes, particularly in executive functions [9]. Beyond overall diet quality, dietary diversity represents another dimension of diet, reflecting the variety of food groups consumed; the two constructs are not interchangeable, since a diet may be varied without being of high quality. The relationship between cognitive function and dietary diversity in older adults has not been fully clarified. A study conducted on older adults living in Japan found an association between high dietary diversity and low cognitive impairment, whereas a study conducted in Türkiye found no association between dietary diversity and cognitive impairment [10,11]. Jiang et al. [12] reported that a higher Chinese Healthy Eating Index score was associated with better cognitive function in older adults, with psychological balance and depressive symptoms acting as chain mediators.

In addition to dietary factors, anthropometric status may be associated with cognitive performance in later life. Studies suggest an association between obesity, measured by body mass index (BMI) and especially waist–hip ratio (WHR) and waist circumference (WC), and lower cognitive performance in older adults [13,14]. However, it remains unclear whether general adiposity (indexed by BMI) and central adiposity (indexed by WC and WHR) provide comparable information on cognitive performance since these indicators are frequently used interchangeably, despite capturing different aspects of body composition. Although research on the relationship between obesity and cognitive impairment in older adults has increased, conflicting results persist [15].

Although evidence regarding the associations of dietary patterns and anthropometric status with cognitive function is increasing, studies that evaluate these factors simultaneously remain limited. This distinction matters because adiposity is itself partly determined by long-term dietary intake; unless diet quality, dietary diversity, and anthropometric indicators are modeled jointly, it cannot be determined whether diet is associated with cognitive performance independently of body size, or whether an apparent dietary association is instead explained by adiposity. Evidence from Türkiye is particularly scarce. Previous studies conducted in Türkiye have examined nutritional status and cognitive function; however, evidence combining dietary diversity, overall diet quality, and multiple anthropometric indicators remains insufficient [16,17]. This setting is relevant because obesity is highly prevalent among older adults in Türkiye, and habitual dietary patterns differ from those of Western cohorts in which most of the available evidence has been generated. Accordingly, this study aimed to examine the relationships of anthropometric status, dietary diversity, and dietary quality with cognitive performance in older adults attending a nutrition and dietetics outpatient clinic in Türkiye. We hypothesized that higher adiposity, lower diet quality, and lower dietary diversity would each be independently associated with poorer cognitive performance after accounting for age, sex, and years of education.

## 2. Materials and Methods

### 2.1. Study Design and Participants

This cross-sectional study was conducted between April 2026 and June 2026 among individuals aged 65 years and older who applied to the Nutrition and Diet Outpatient Clinic of Kastamonu Physical Therapy and Rehabilitation Hospital. The sample size was calculated using G*Power 3.1.9.7 (Heinrich Heine University Düsseldorf, Düsseldorf, Germany). An a priori power analysis for multiple linear regression was performed with the following parameters: a medium effect size (f^2^ = 0.15), a significance level of α = 0.05, statistical power of 1 − β = 0.80, and seven independent variables. The analysis indicated that at least 103 participants should be included in the study. This study used a non-probability sequential sampling method.

This study included individuals aged 65 years and older who voluntarily participated, were able to complete cognitive tests and nutritional assessments, had no acute health condition preventing measurements, and who applied to the Nutrition and Diet Outpatient Clinic of Kastamonu Physical Therapy and Rehabilitation Hospital. Individuals with physician-diagnosed dementia (including Alzheimer’s disease), psychiatric disorders, or a history of acute infection, surgery, major trauma, or hospitalization within the past two weeks were excluded.

The study was approved by the Non-Interventional Clinical Research Ethics Committee of Kastamonu University (Approval No.: 2026-63, date: 19 March 2026) and was conducted in accordance with the Declaration of Helsinki. Institutional permission to conduct this research at the Kastamonu Physical Therapy and Rehabilitation Hospital was obtained from the Kastamonu Provincial Health Directorate (Document number: E-44008972-770, date: 25 February 2026). Additionally, this article was prepared in accordance with the STROBE checklist.

### 2.2. Data Collection

A face-to-face questionnaire has six sections. Section 1 included information on participants’ sociodemographic characteristics (age, gender, marital status, education level, and cohabitation status). Section 2 included anthropometric measurements such as body weight, height, waist and hip circumferences, along with body fat analysis results. Section 3 gathered information on health status, including the presence of chronic diseases, appetite, dietary habits, and smoking and alcohol use. Section 4 assessed participants’ food intake using 24 h dietary recall. Section 5 included cognitive performance, which was assessed using the Montreal Cognitive Assessment (MoCA). Section 6 assessed physical activity level using the International Physical Activity Questionnaire Short Form (IPAQ-SF).

Participants’ educational information was collected self-report during face-to-face interviews. Educational level was determined by asking participants to indicate the most recent level of education had completed and from which they had received a diploma; it was classified as primary school, secondary/high school, and university graduate. The number of years of education was recorded as the total duration of formal education completed.

### 2.3. Anthropometric Measurements

Height was measured using a portable stadiometer (Seca 213, seca GmbH & Co. KG, Hamburg, Germany), with participants barefoot and wearing light clothing. Body weight and body composition were assessed using a Tanita BC-401 bioelectrical impedance analysis device (Tanita Corporation, Tokyo, Japan). Measurements were taken under standardized conditions between 08:00 and 10:00 a.m. following an overnight fast of at least 12 h. Participants were instructed to avoid vigorous physical activity and alcohol consumption for 24 h and to avoid caffeine intake for at least 12 h prior to the assessment. They were also asked to void their bladder immediately before the measurement and were assessed barefoot, wearing light clothing, with all metal accessories removed [18]. BMI was calculated as body weight (kg) divided by height squared (m^2^). Waist and hip circumferences were measured using a non-elastic tape measure; WC was measured at the midpoint between the last rib and the iliac crest, and hip circumference was measured at the widest part of the greater trochanter. WHR was calculated as waist circumference divided by hip circumference, with substantially increased risk defined as ≥0.90 for men and ≥0.85 for women [19].

### 2.4. Dietary Intake

Dietary intake was assessed using the 24 h dietary recall method for one weekday. Participants were asked to recall and report in detail all foods and beverages consumed during the previous 24 h. Nutrition Photo Catalog Measurements and Amounts were used to ensure accurate reporting of portion size [20]. Energy and nutrient intake for older adults were analyzed using the BEBIS (version 9.0) software [21]. Dietary diversity and dietary quality scores were calculated using data obtained from participants’ 24 h retrospective food intake records.

### 2.5. Dietary Diversity

The evaluation of dietary diversity was based on the Food and Agriculture Organization of the United Nations [22]. According to this report, the Dietary Diversity Score (DDS) is defined as the number of food groups consumed within 24 h. The diet was classified according to the nine food groups recommended by the FAO: (1) starchy staples; (2) dark green leafy vegetables; (3) vitamin A-rich fruits and vegetables; (4) other fruits and vegetables; (5) organ meats; (6) meat, poultry, and fish; (7) eggs; (8) legumes, nuts, and seeds; and (9) milk and milk products. Tea, sugar, and sweets were not included in the DDS calculations. Participants were categorized according to their DDS as follows: poorly diversified diet (DDS 0–3), moderately diversified diet (DDS 4–5), and diversified diet (DDS 6–9).

### 2.6. Diet Quality

Diet quality was assessed using the Healthy Eating Index-2020 (HEI-2020), which evaluates adherence to the 2020–2025 Dietary Guidelines for Americans. HEI-2020 scores were calculated from dietary intake records using the simple scoring algorithm. Intakes of the food group components were expressed per 1000 kcal of energy intake (energy-density approach), fatty acids were evaluated as the ratio of unsaturated to saturated fatty acid; and added sugars and saturated fats were expressed as percentages of total energy intake. The HEI-2020 includes 13 components, grouped into two categories: adequacy (total fruits, whole fruits, total vegetables, greens and beans, whole grains, dairy, total protein foods, seafood and plant proteins, and fatty acids) and moderation (refined grains, sodium, added sugars, and saturated fats). Each component is scored based on intake levels: adequacy components receive higher scores for greater consumption, while moderation components receive higher scores for lower consumption. The total HEI-2020 score ranges from 0 to 100, with higher scores reflecting better diet quality [23].

### 2.7. Montreal Cognitive Assessment

The MoCA, developed to evaluate mild cognitive impairment, assesses various cognitive domains including executive functions, visuospatial skills, memory, language, attention and concentration, abstract thinking, calculation, and orientation. In the original study, the MoCA had good internal consistency, and the Cronbach’s alpha value for the standardized items was found to be 0.83 [24]. The MoCA was adapted into Turkish and validated by Selekler et al. The Cronbach’s alpha value for the MoCA is not reported in the Turkish version [25]. The maximum total score is 30. The education-specific cut-off scores proposed by Kaya et al. [26] were used to classify cognitive performance. Participants with MoCA scores below 18 among those with ≤5 years of education, below 21 among those with 6–11 years of education, and below 23 among those with ≥12 years of education were classified as screening positive for possible MCI. Scores at or above the respective cut-off values were classified as normal cognitive performance. In the study conducted by Kaya et al. [26], the Cronbach’s alpha value of the MoCA was 0.81. In this study, the Cronbach’s alpha for the MoCA was 0.80. The MoCA was administered face-to-face by trained researchers using the validated Turkish version. Because education-specific cut-off points were used, the standard one-point adjustment for participants with 12 or fewer years of education was not applied. These education-specific thresholds were preferred over a single universal cut-off because formal education has been shown to substantially influence MoCA performance in the Turkish population.

### 2.8. Physical Activity

The IPAQ-SF was designed to assess physical activity and sedentary behavior in adults, and is available in both short and long versions. Its international validity and reliability were established by Craig et al. [27], and the Turkish adaptation was validated by Saglam et al. [28]. In this questionnaire, physical activities were required to be performed for at least 10 min at a time. Participants were asked about vigorous and moderate physical activity, walking, and daily sitting time over the past seven days. The durations of vigorous, moderate, and walking activities were converted to metabolic equivalents (METs; 1 MET = 3.5 mL/kg/min) using the following calculations: MET-min/week scores for walking, moderate, and vigorous physical activity were calculated by multiplying the MET coefficient for each activity (3.3, 4.0, and 8.0) by the activity duration and the number of days per week. The total physical activity score (MET-min/week) was obtained by summing the walking, moderate, and vigorous activity scores. Based on the TPAS, physical activity levels were classified as follows: TPAS < 600 MET-min/week was considered low physical activity; TPAS of 600–3000 MET-min/week was considered moderate physical activity; and TPAS > 3000 MET-min/week was considered high physical activity.

### 2.9. Statistical Analysis

The data obtained in the study were analyzed using IBM SPSS Statistics 25.0 (IBM Corp., Armonk, NY, USA). Descriptive statistics, including the arithmetic mean, standard deviation, median, minimum, maximum, percentages, and frequency distributions, were used to analyze the data. In this study, MoCA score was considered as the dependent variable, while age, sex, years of education, physical activity, hypertension, diabetes mellitus, hypercholesterolemia, cardiovascular disease, anthropometric measurements, DDS, and HEI-2020 score were evaluated as independent variables. The normality of the variables was assessed using the Kolmogorov–Smirnov test and Q–Q plots; histograms were also used for visual inspection. Homogeneity of variance was tested for group comparisons using Levene’s test. For comparisons between two groups, Student’s *t*-test was applied to normally distributed variables, while the Mann–Whitney U test was applied to non-normally distributed variables. Categorical variables were compared using Pearson’s chi-squared test. When expected cell frequencies were insufficient, Fisher’s exact test was applied. When an overall association was significant for variables with more than two categories, post hoc comparisons were performed using adjusted standardized residuals with Bonferroni correction. Spearman’s correlation analysis was used to examine the relationships and strengths among numerical variables. Hierarchical regression analysis was performed to identify factors associated with cognitive performance among older adults. Variables were entered into the primary hierarchical regression in three blocks: age, sex, years of education, and physical activity; BMI; and DDS and HEI-2020. Sensitivity analyses additionally adjusted the primary model for hypertension, diabetes mellitus, hypercholesterolemia, and cardiovascular disease and, in separate models, replaced BMI with WC or WHR. After the addition of comorbidities to the model, the change in the magnitude and statistical significance of the relationship between BMI and MoCA score was examined to evaluate the sensitivity of the results to these potential confounding variables. Furthermore, the contribution of the comorbidity block to the model was assessed using the change in explained variance (ΔR^2^) and its significance test. Before interpretation, the assumptions of linear regression were examined: linearity and homoscedasticity, assessed by inspection of residual plots; normality of residuals, assessed using normal probability plots; independence of residuals, assessed using the Durbin–Watson statistic; and multicollinearity, assessed using tolerance and variance inflation factors. All analyses were performed on complete cases; participants with missing data were excluded before analysis. All statistical tests were two-sided. A nominal *p*-value < 0.05 was considered statistically significant unless otherwise specified. No global adjustment for multiple testing was applied across the remaining analyses; therefore, the findings from these analyses, particularly the BMI–MoCA association, were interpreted as exploratory.

## 3. Results

### 3.1. Participant Characteristics

During the study period, 881 older adults attended the Nutrition and Diet Outpatient Clinic. Of these, 441 did not meet the eligibility criteria. Among the 440 eligible individuals, 317 declined to participate and 123 were enrolled. Six participants were subsequently excluded because of missing or incomplete data, resulting in a final analytic sample of 117 participants. A total of 117 older adults, of whom 36 were men and 81 were women, were assessed. The sociodemographic, health, lifestyle, cognitive, and body mass index characteristics of the participants according to sex are presented in Table 1. Accordingly, the percentage of married individuals was higher in men than in women (91.7% vs. 65.4%, respectively; *p* < 0.05). However, the percentage of participants living alone was higher in women than in men (25.9% vs. 2.8%, respectively; *p* < 0.05). Furthermore, the prevalence of hypertension was found to be higher in women than in men (70.4% vs. 47.2%; *p* < 0.05). Physical activity levels differed significantly between men and women (*p* = 0.031), with a higher proportion of women having low physical activity levels than men (79.0% vs. 55.6%). The percentage of participants with obesity was higher in women than in men (65.4% vs. 25.0%; *p* < 0.01). There were no statistically significant differences between the groups in median age, education level, chronic disease prevalence, smoking status, appetite, meal frequency, and MoCA classification (*p* > 0.05).

### 3.2. Sociodemographic, Health, Lifestyle, and Physical Activity According to Cognitive Performance

The sociodemographic, health, lifestyle, and physical activity characteristics of the participants, according to the MoCA classification, are presented in Table 2. The participants who screened positive for possible MCI differed significantly from those with normal cognitive performance with respect to education level, appetite status, and number of snacks. Primary school education was more common in the possible MCI group (93.5% vs. 74.5%, *p* = 0.016). Snack frequency differed significantly between groups (*p* < 0.001); not snacking was more common in the group with normal cognitive performance, whereas consuming two snacks was more common in the possible MCI group. Appetite status differed significantly between the groups (*p* = 0.019). Normal appetite was more common among participants screening positive for possible MCI than among those with normal cognitive performance (35.5% vs. 16.4%). No significant differences were observed for age, sex, marital status, living arrangement, chronic disease status, specific chronic diseases, smoking, number of main meals, and physical activity level.

### 3.3. Anthropometric and Dietary Characteristics According to Cognitive Performance

Anthropometric measurements and dietary characteristics according to the MoCA classification are presented in Table 3. Participants who screened positive for possible MCI had a significantly higher mean BMI than those with normal cognitive performance (32.67 ± 5.78 vs. 29.54 ± 3.39 kg/m^2^, *p* < 0.001). Energy intake per kilogram of body weight was significantly lower in the possible MCI group than in the normal cognitive performance group (19.26 (14.85–25.46) vs. 22.64 (18.87–28.80) kcal/kg/day, *p* = 0.029). No significant differences were observed between the groups for body fat percentage, WC, WHR, DDS, HEI-2020 score, total energy intake, total protein intake, protein intake per kilogram of body weight, or percentage of energy derived from protein (all *p* > 0.05). When BMI was dichotomized as non-obese (<30.0 kg/m^2^) and obese (≥30.0 kg/m^2^), the prevalence of obesity was significantly higher in the possible MCI group than in the normal cognitive performance group (62.9% vs. 41.8%; Fisher’s exact test, *p* = 0.027).

### 3.4. Correlations with MoCA Scores

Spearman correlations among MoCA score, anthropometric measurements, nutrient intakes, and diet quality indicators are presented in Table 4. A significant negative correlation was found between MoCA scores and BMI (r = −0.303, *p* = 0.001); higher BMI values were associated with lower MoCA scores. No statistically significant correlations were observed between the MoCA score and body fat percentage, WC, WHR, DDS, HEI-2020 score, total or body weight-adjusted energy intake, total or body weight-adjusted protein intake, or percentage of energy derived from protein (all *p* > 0.05).

### 3.5. Factors Associated with MoCA Scores

Table 5 presents the hierarchical multiple linear regression analysis of sociodemographic, physical activity, anthropometric, and dietary factors associated with MoCA scores in the participants. Model 1, which included age, sex, years of education, and physical activity, was statistically significant and explained 19.7% of the variance in MoCA scores (R^2^ = 0.197, adjusted R^2^ = 0.168; F(4, 112) = 6.864, *p* < 0.001). Adding BMI (Model 2) significantly increased the explained variance (ΔR^2^ = 0.042, ΔF = 6.060, *p* = 0.015). Although the inclusion of DDS and HEI-2020 in Model 3 increased the explained variance by an additional 2.1%, this increase was not statistically significant (ΔR^2^ = 0.021, ΔF = 1.558, *p* = 0.215). The final model was statistically significant and explained 26.0% of the total variance in MoCA scores (R^2^ = 0.260, adjusted R^2^ = 0.212; F(7, 109) = 5.460, *p* < 0.001). In the final model, more years of education were associated with higher MoCA scores (B = 0.768, β = 0.363, 95% CI: 0.412 to 1.124, *p* < 0.001). In contrast, higher BMI continued to be associated with lower MoCA scores (B = −0.301, β = −0.240, 95% CI: −0.527 to −0.074, *p* = 0.010). Age, sex, physical activity, dietary diversity, and HEI-2020 scores were not significantly associated with MoCA scores. The adjusted R^2^ of the final model was 0.212, suggesting that a significant portion of the variance in cognitive performance may be attributable to other factors not included in the model. Regression diagnoses did not indicate problematic multicollinearity (tolerance = 0.816–0.953; VIF = 1.050–1.226). The Durbin–Watson statistic was found to be 1.689, and all Cook distance values were below 1 (maximum = 0.599). A visual inspection of the graph of standardized residuals and predicted values showed that the assumptions of linearity and homoskedasticity were reasonably met.

### 3.6. Sensitivity Analyses

Sensitivity analyses examining alternative anthropometric indicators and additional adjustment for comorbidities are presented in Table 6. The explained variance increased by 4.2% after the addition of BMI to the model (ΔR^2^ = 0.042, *p* = 0.015). In contrast, neither WC nor WHR significantly increased the explained variance (ΔR^2^ = 0.007, *p* = 0.342; ΔR^2^ = 0.002, *p* = 0.640, respectively). The dietary block did not significantly improve any of the models. Neither dietary diversity nor HEI-2020 was significantly associated with MoCA scores. In an additional sensitivity analysis to assess the robustness of the findings, the final BMI model was further adjusted for diabetes mellitus, hypertension, hypercholesterolemia, and cardiovascular disease. Adding the comorbidity block did not significantly increase the explained variance (ΔR^2^ = 0.014, *p* = 0.762). However, the inverse association between BMI and MoCA scores remained significant (B = −0.305, β = −0.243, *p* = 0.011).

## 4. Discussion

### 4.1. Principal Findings

This study investigated the relationships between anthropometric status, dietary diversity, and nutritional quality, and cognitive performance in older Turkish adults. Most participants (53.0%) had a positive screening result for possible mild cognitive impairment according to the MoCA. One of the main findings of the study is that higher BMI was associated with lower MoCA scores in the primary model (which included age, sex, years of education, physical activity, BMI, DDS, and HEI-2020) and this association remained significant after additional adjustment for hypertension, diabetes mellitus, hypercholesterolemia, and cardiovascular disease. In contrast, waist circumference and waist-to-hip ratio, which were used in the sensitivity analyses, did not show a significant association with cognitive performance. Furthermore, years of education were found to be the strongest independent factor associated with MoCA scores, with more years of education being associated with better cognitive performance. However, no significant relationship was found between single 24 h dietary measures (DDS and HEI-2020), which reflect dietary diversity and nutritional quality, and MoCA scores in this sample. Failure to detect a relationship between diet quality and cognitive performance in this study does not definitively prove that a true relationship is absent. However, the DDS and HEI-2020 results should be interpreted with caution. Both indicators are calculated from a single 24 h dietary recall and therefore may not fully reflect participants’ usual dietary patterns. Daily individual variability may lead to measurement error, especially for the HEI-2020, which aims to assess long-term diet quality. Consequently, the lack of a significant relationship between DDS and HEI-2020 and MoCA scores does not imply the absence of a real relationship between diet quality and cognitive performance. Measurement error and variability may have contributed to the weakening of the relationships. Although the final model was statistically significant, its explanatory capacity was modest (R^2^ = 0.260; adjusted R^2^ = 0.212), indicating that a significant portion of the variability in MoCA scores was not captured by the variables included in the model. Accordingly, the observed relationships for BMI and education should not be interpreted as a comprehensive explanation of cognitive performance.

### 4.2. Cognitive Performance and Educational Factors

Among the older adults participating in the present study (median age: 71 years), 53.0% screened positive for possible MCI based on the MoCA. A rate of 70.9% was reported in the study conducted by Ye et al. [29] (mean age: 71.18 ± 4.97 years) and 35.6% was reported in the study conducted by Wu et al. [30] (mean age: 68.9 years). In the study by Ye et al. [29], the use of a higher MoCA cut-off point (<26) may have contributed to the higher proportion of participants screening positive for possible MCI. Similar to the present study, Wu et al. [30] used education-specific MoCA cut-off points. However, the hospital-based design of the present study, the lower educational level of our sample, and its higher mean age may have contributed to the higher proportion of participants screening positive for possible MCI. Differences in countries, cultures, health status, and sampling methods may produce different results when cognitive performance is assessed. In this study, years of education showed the strongest independent association with MoCA scores, with a longer duration of education being associated with better cognitive performance. In addition, a low level of education was more prevalent in the possible MCI group. Our findings are consistent with previous studies reporting positive correlations between MoCA scores and duration of education [29,31]. However, the association between education and higher MoCA scores may not be attributable entirely to better cognitive health; the sensitivity of the MoCA to educational level may also partially explain this association [32]. However, 84.6% of the participants in this study had received primary education; this indicates that the sample is predominantly characterized by a low level of formal education. This educational profile is particularly important when interpreting the 53.0% of participants who screened positive for possible MCI. To account for the known effect of education on MoCA performance, education-specific threshold scores validated for the Turkish population were used instead of a single universal threshold [26]. However, education-specific thresholds may not completely eliminate the effect of formal education on MoCA performance. The strong positive association between years of education and MoCA scores observed in the adjusted analysis may therefore reflect both differences in cognitive performance and residual effects of education on the assessment. Accordingly, the proportion of those who had a positive result in the screening for possible mild cognitive impairment according to the education level-specific Turkish criteria may have been influenced by the sample’s predominantly low educational level.

### 4.3. Anthropometric Status and Cognitive Performance

The negative association between BMI and MoCA scores remained significant after controlling for age, sex, years of education, physical activity, common comorbidities, and dietary indicators in our study. Furthermore, participants with obesity had significantly lower MoCA scores than those without obesity. In contrast, neither WC nor WHR was associated with MoCA scores in the sensitivity analyses. The lack of consistency across anthropometric indicators suggests that the observed association may be measure-specific and should not be interpreted as evidence of a robust association between adiposity and cognitive performance. Although Phirom et al. [15] found that BMI, WC, and WHR may be associated with poor cognitive performance, they reported that the available evidence was of low certainty and highly uncertain. Another study conducted among older adults observed a positive linear association between WC and cognitive impairment but found no significant association with BMI [33]. Another study based on data from the China Health and Retirement Longitudinal Study reported an association between BMI and cognitive impairment. Being underweight was identified as a risk factor for the development of cognitive impairment, whereas being overweight or obese could reduce the likelihood of cognitive impairment, particularly among older women [34]. In contrast, Alvarez et al. [35] reported that higher WC and BMI were associated with cognitive impairment assessed using the MoCA. These differences across studies suggest that the relationship between anthropometric indicators and cognitive performance in older adults is complex and may vary according to the indicator used, sample characteristics, and comorbid health conditions. Several biological mechanisms have been proposed to explain the observed association between higher BMI and decreased cognitive performance, including chronic inflammation and metabolic dysregulation. Some studies have suggested that the relationship between obesity and cognitive performance may be linked to co-occurring health conditions such as hypertension, cardiovascular disease, and diabetes mellitus [36]. However, some alternative biological and methodological explanations should also be considered when interpreting the relationship between BMI and cognitive performance. First, BMI may not fully reflect body composition in older adults; conditions such as sarcopenic obesity, where decreased muscle mass and increased fat mass occur together, may be associated with cognitive health beyond effects solely from body weight. Recent systematic reviews and meta-analyses have shown that sarcopenic obesity is associated with an increased risk of cognitive dysfunction and dementia [37,38]. Second, lifetime changes in body weight and the effects of obesity on cognitive health in middle and late life may differ. Longitudinal studies report that the relationship between adiposity and cognitive decline may vary depending on age, the direction of weight change, and the life stage assessed [39,40]. Furthermore, due to the cross-sectional design of our study, the possibility of reverse causality cannot be ruled out; early cognitive decline may have contributed to the observed relationship by influencing physical activity levels, dietary habits, and body weight. Indeed, previous studies highlight the potentially bidirectional relationship between obesity and cognitive function [41,42]. Finally, the possibility of residual confounding cannot be entirely ruled out, as depressive symptoms, frailty status, and other unmeasured health factors may be associated with both BMI and cognitive performance. In particular, the relationship between obesity and frailty, as well as the close association of frailty with cognitive impairment, may complicate the interpretation of the BMI–cognition relationship observed in older adults [43,44]. Similarly, in the present study, the prevalence of comorbidities did not differ between the MoCA groups. The increasing prevalence of both obesity and cognitive performance problems worldwide and in Türkiye [1] necessitates a more detailed examination of the relationship between these two conditions and the elucidation of their causal aspects through longitudinal studies. However, the observed relationship with BMI requires careful interpretation. A significant association with cognitive performance was observed only for BMI; similar results were not obtained for other anthropometric indicators such as waist circumference and waist-to-hip ratio. If the observed relationship directly reflects the effect of adiposity on cognitive performance, more consistent findings could be expected among different indicators of adiposity. Therefore, it is possible that the relationship between BMI and MoCA scores reflects different aspects of body composition, that BMI is a flawed indicator of adiposity in older adults, or that it is affected by random variability due to sampling. While our results suggest that BMI may be associated with cognitive performance, this relationship needs to be confirmed with other anthropometric measures.

In an additional sensitivity analysis, the primary model was further adjusted for hypertension, diabetes mellitus, hypercholesterolemia, and cardiovascular disease to account for potential confounding by these comorbid conditions. The association between BMI and MoCA scores remained statistically significant after this additional adjustment. Nevertheless, residual confounding cannot be excluded, as depressive symptoms, medication use, frailty status, and other unmeasured factors may be associated with both BMI and cognitive performance. Therefore, the observed relationship between BMI and cognitive performance should not be interpreted as causal or fully independent of other factors. Given the absence of a global adjustment for multiple testing, the association between BMI and MoCA scores should be interpreted as exploratory and requires confirmation in independent studies.

### 4.4. Dietary Diversity and Cognitive Performance

In this study, no significant association was found between DDSs, an indicator of dietary diversity, and MoCA scores. Previous studies have reported that low dietary diversity is associated with poorer cognitive performance [45,46]. Increasing dietary diversity, which refers to the variety or number of different food groups consumed over a given period, may contribute to the maintenance of brain health by supporting adequate nutrient intake and may potentially play a role in reducing the risk of neurodegenerative diseases [46]. However, contrary to some findings in the literature, dietary diversity was not associated with cognitive performance in the present study. This discrepancy may be attributable to differences in the methods used to calculate dietary diversity, the cognitive assessment instruments employed, and sample characteristics. Furthermore, assessing dietary intake using a single-day dietary record may not have fully reflected the participants’ usual dietary diversity. The relatively high DDSs and limited variability among the participants may also have reduced the ability to detect a possible association.

### 4.5. Diet Quality and Cognitive Performance

In the present study, no significant association was detected between the HEI-2020 score derived from the single 24 h dietary assessment and MoCA scores. Using data from the Microbiome in Aging Gut and Brain (MiaGB) Consortium Cohort, Arikawa et al. [47] examined the associations of the inflammatory potential of the diet and overall diet quality with cognitive impairment. The researchers reported no significant differences in Dietary Inflammatory Index or HEI-2020 scores between participants with and without cognitive impairment. Similarly, another study examining the associations of the Mediterranean–DASH Intervention for Neurodegenerative Delay (MIND) diet and HEI-2020 with cognitive performance found that higher MIND diet scores were associated with better global cognitive performance and memory, whereas HEI-2020 scores were not associated with global cognition, memory, or executive function [48]. In contrast, Wessinger et al. [49] reported that higher HEI-2020 scores were associated with better executive function and working memory among participants who were not carriers of the APOE-ε4 allele, whereas no significant association was observed among APOE-ε4 carriers. These findings suggest that the association between overall diet quality and specific cognitive domains may vary according to genetic susceptibility. Another study conducted among middle-aged and older adults also evaluated the relationship between cognitive function and healthy lifestyle components (diet quality, physical activity, and smoking). This research reported that higher levels of physical activity were associated with higher scores in overall cognitive performance and some cognitive domains, and that individuals with healthy lifestyle characteristics performed better cognitively. Although this study considered lifestyle factors as a whole and also evaluated physical activity, the results support the importance of nutrition and other lifestyle behaviors in maintaining cognitive health. In another study conducted among middle-aged and older adults in Saudi Arabia, Naaman et al. [50] reported that good diet quality was associated with higher scores in the naming cognitive domain. However, diet quality was not significantly associated with overall cognitive performance. The fact that the HEI-2020 was developed to assess adherence to general healthy eating recommendations rather than as an index specifically designed for cognitive health may be one possible explanation for the absence of a significant association between it and cognitive performance in the present study [23].

In this study, no statistically significant association was found between DDSs and HEI-2020 scores calculated from a single 24 h dietary recall and MoCA scores. However, this finding should not be interpreted as there being no relationship between dietary quality or dietary variety and cognitive performance. Since both indicators are based on a single-day food intake record, they may not fully reflect the usual dietary patterns of the participants. For the HEI-2020, which aims to assess long-term dietary quality, daily individual variability can lead to measurement error and weaken potential associations.

### 4.6. Energy and Protein Intake and Cognitive Performance

In comparisons of dietary characteristics between the groups, energy intake per kilogram of body weight was higher among participants with normal cognitive performance (22.64 kcal/kg/day) than among those in the possible MCI group (19.26 kcal/kg/day). However, no significant differences were found between the groups in terms of total energy or protein intake. Similarly, Doorduijn et al. [51] reported no significant differences in total energy, protein, carbohydrate, or fat intake between older adults with MCI and controls. However, Park et al. [52] showed that after controlling for confounding factors, older adults with energy intakes below the recommended level were more likely to have cognitive impairment than those whose energy intakes met the recommended level. Although these findings indicate the potential importance of adequate energy intake in maintaining cognitive health, further studies are needed to clarify the relationship between energy intake and cognitive decline in older adults and the potential effects of energy restriction. In the present study, good appetite was more prevalent in the normal cognitive performance group than in the possible MCI group, whereas normal appetite was more prevalent in the possible MCI group. This finding suggests that good appetite may be associated with normal cognitive performance. Similarly, a study conducted among individuals with diabetes mellitus aged over 60 years found that participants with cognitive impairment were more likely to report a reduced appetite [53]. In contrast, no significant association between poor appetite and cognitive function was observed among community-dwelling individuals aged 55 years and older [54]. Previous research highlights the importance of the interaction between cognitive health and lifestyle factors. A study of community-dwelling older adults in China showed that the protective effect of physical exercise on cognitive impairment varied depending on certain dietary habits, such as the consumption of fruits, dairy products, and legumes. While differing from our study in terms of research design and evaluated variables, these findings support the idea that nutritional status may be related to cognitive health in older individuals [55]. Differences across studies may be attributable to the age and health characteristics of the samples, the methods used to assess appetite, and the confounding factors controlled for in the analyses.

### 4.7. Strengths and Limitations

This study has several limitations. First, due to its cross-sectional design, the relationships between anthropometric indicators, nutritional characteristics, and cognitive performance cannot be interpreted causally. Reverse causality cannot be ruled out, as lower cognitive performance is likely influenced by dietary behaviors, physical activity, and body weight. Second, the study’s single-center design, reliance on a relatively small sample size, and high rejection rate among eligible participants may have contributed to selection bias and limited the generalizability of the findings. Individuals who agreed to participate may have differed from those who declined in terms of health status, healthcare-seeking behaviors, dietary habits, or cognitive status. Third, dietary intake was assessed using a single 24 h recall. This may not fully reflect participants’ usual dietary habits and could have led to weaker associations between DDS and HEI-2020 and cognitive performance. Fourth, physical activity was assessed based on self-report, and cognitive status was determined using MoCA rather than clinical diagnosis. Therefore, participants classified as screening positive for possible MCI in this study should not be considered to have clinically confirmed MCI. Finally, the possibility of residual confounding cannot be entirely ruled out. However, the fact that the association between BMI and MoCA scores remained significant in additional sensitivity analyses performed for diabetes mellitus, hypertension, hypercholesterolemia, and cardiovascular disease indicates that the findings are robust against these important comorbidities. However, other potential confounding variables such as depressive symptoms, medication use, disease duration, and disease severity were not evaluated. Therefore, it cannot be said that the observed association is completely independent of all possible confounding factors.

Nevertheless, the study has significant strengths. The use of validated education level-specific MoCA threshold values for the Turkish elderly population, the assessment of nutrition using both DDS and HEI-2020, and the performance of sensitivity analyses including alternative anthropometric indicators and comorbidity adjustments have increased the reliability of the findings. Furthermore, hierarchical regression analyses and sensitivity analyses using alternative anthropometric indicators allowed the consistency of the findings across different measures of adiposity to be evaluated. The findings need to be confirmed by data from different regions and community-based samples and validated by longitudinal studies.

## 5. Conclusions

In conclusion, obesity was more common among participants who screened positive for possible MCI than among those with normal cognitive performance, and a higher BMI was associated with lower MoCA scores. However, this association was not observed for other anthropometric indicators such as waist circumference and waist-to-hip ratio. Therefore, the finding should be interpreted cautiously and confirmed by larger, longitudinal studies using different adiposity measures. Dietary diversity and HEI-2020 scores were not associated with MoCA scores. No significant association was observed between dietary quality indicators based on a single 24 h recall and cognitive performance; however, this finding should not be interpreted as there being no relationship between dietary quality and cognitive performance. The number of years of education showed a strong positive association with cognitive performance. Longitudinal studies using repeated dietary assessments and more comprehensive measures of body composition are needed to clarify these relationships.

## Figures and Tables

**Table 1:** Sociodemographic, health, lifestyle, cognitive, and body mass index characteristics of older adults according to sex.

Variable	Category	Male (*n* = 36), *n* (%)	Female (*n* = 81), *n* (%)	Total (*n* = 117), *n* (%)	*p*-Value
Age, years, median (Q1–Q3)		71 (69.3–76.0)	72 (69.0–76.0)	71 (69.5–76.0)	0.991 ^2^
Education level	Primary school	28 (77.8)	71 (87.7)	99 (84.6)	0.200 ^1^
	Secondary and high school	6 (16.7)	5 (6.2)	11 (9.4)	
	University	2 (5.6)	5 (6.2)	7 (6.0)	
Marital status	Married	33 (91.7)	53 (65.4)	86 (73.5)	**0.003** ^1^
	Not married	3 (8.3)	28 (34.6)	31 (26.5)	
Living arrangement	Living with family	35 (97.2)	60 (74.1)	95 (81.2)	**0.003** ^1^
	Living alone	1 (2.8)	21 (25.9)	22 (18.8)	
Chronic disease	Yes	31 (86.1)	69 (85.2)	100 (85.5)	0.896 ^1^
	No	5 (13.9)	12 (14.8)	17 (14.5)	
Specific chronic diseases	Hypertension	17 (47.2)	57 (70.4)	74 (63.2)	**0.017** ^1^
	Hypercholesterolemia	6 (16.7)	13 (16.0)	19 (16.2)	0.933 ^1^
	Cardiovascular disease	7 (19.4)	15 (18.5)	22 (18.8)	0.906 ^1^
	Diabetes mellitus	20 (55.6)	33 (40.7)	53 (45.3)	0.137 ^1^
	Gastrointestinal diseases	1 (2.8)	0 (0)	1 (0.9)	0.132 ^1^
	Prostate disease	4 (11.1)	0 (0)	4 (3.4)	–
Smoking status	Yes	3 (8.3)	2 (2.5)	5 (4.3)	0.148 ^1^
	No	33 (91.7)	79 (97.5)	112 (95.7)	
Alcohol consumption	No	36 (100)	81 (100)	117 (100)	–
Appetite status	Normal	25 (69.4)	61 (75.3)	86 (73.5)	0.507 ^1^
	Good	11 (30.6)	20 (24.7)	31 (26.5)	
Number of main meals	2	12 (33.3)	39 (48.1)	51 (43.6)	0.136 ^1^
	3	24 (66.7)	42 (51.9)	66 (56.4)	
Number of snacks	None	9 (25.0)	26 (32.1)	35 (29.9)	0.844 ^1^
	1	10 (27.8)	21 (25.9)	31 (26.5)	
	2	12 (33.3)	26 (32.1)	38 (32.5)	
	3	5 (13.9)	8 (9.9)	13 (11.1)	
Physical activity level	Low	20 (55.6)	64 (79.0)	84 (71.8)	**0.031** ^1^
	Moderate	14 (38.9)	16 (19.8)	30 (25.6)	
	High	2 (5.6)	1 (1.2)	3 (2.6)	
MoCA classification	Possible MCI	17 (47.2)	45 (55.6)	62 (53.0)	0.429 ^1^
	Normal cognitive performance	19 (52.8)	36 (44.4)	55 (47.0)	
BMI classification	Normal, 18.5–24.9 kg/m^2^	5 (13.9)	4 (4.9)	9 (7.7)	**<0.001** ^1^
	Overweight, 25.0–29.9 kg/m^2^	22 (61.1)	24 (29.6)	46 (39.3)	
	Obese, ≥30.0 kg/m^2^	9 (25.0)	53 (65.4)	62 (53.0)	

^1^ Chi-square test; ^2^ Mann–Whitney U test. Bold values indicate statistical significance (*p* < 0.05). BMI, body mass index; possible MCI, screening positive for possible mild cognitive impairment; MoCA, Montreal Cognitive Assessment; Q1–Q3, first to third quartiles. Education-specific MoCA cut-off scores were applied: <18 for participants with ≤5 years of education, <21 for those with 6–11 years of education, and <23 for those with ≥12 years of education. Scores below the respective cut-off indicate a positive screen for possible mild cognitive impairment.

**Table 2:** Sociodemographic, health, lifestyle, and physical activity characteristics of older adults according to MoCA classification.

Variable	Category	Possible MCI (*n* = 62, 53.0%), *n* (%)	Normal Cognitive Performance (*n* = 55, 47.0%), *n* (%)	*p*-Value ^1^
Age, years, median (Q1–Q3)		71.0 (68.0–76.0)	72.0 (70.0–76.0)	0.332
Sex	Male	17 (27.4)	19 (34.5)	0.405
	Female	45 (72.6)	36 (65.5)	
Education level	Primary school	58 (93.5) ^a^	41 (74.5) ^b^	**0.016**
	Secondary and high school	2 (3.2) ^a^	9 (16.4) ^b^	
	University	2 (3.2) ^a^	5 (9.1) ^a^	
Marital status	Married	45 (72.6)	41 (74.5)	0.810
	Not married	17 (27.4)	14 (25.5)	
Living arrangement	Living with family	50 (80.6)	45 (81.8)	0.871
	Living alone	12 (19.4)	10 (18.2)	
Chronic disease	Yes	54 (87.1)	46 (83.6)	0.596
	No	8 (12.9)	9 (16.4)	
Specific chronic diseases	Hypertension	39 (62.9)	35 (63.6)	0.935
	Hypercholesterolemia	9 (14.5)	10 (18.2)	0.592
	Cardiovascular disease	14 (22.6)	8 (14.5)	0.267
	Diabetes mellitus	28 (45.2)	25 (45.5)	0.975
Smoking status	Yes	4 (6.5)	1 (1.8)	0.369
	No	58 (93.5)	54 (98.2)	
Alcohol consumption	No	62 (100.0)	55 (100.0)	–
Appetite status	Good	40 (64.5) ^a^	46 (83.6) ^b^	**0.019**
	Normal	22 (35.5) ^a^	9 (16.4) ^b^	
Number of main meals	2	29 (46.8)	22 (40.0)	0.461
	3	33 (53.2)	33 (60.0)	
Number of snacks	None	7 (11.3) ^a^	28 (50.9) ^b^	**<0.001**
	1	20 (32.3) ^a^	11 (20.0) ^a^	
	2	27 (43.5) ^a^	11 (20.0) ^b^	
	3	8 (12.9) ^a^	5 (9.1) ^a^	
Physical activity level	Low	42 (67.7)	42 (76.4)	0.118
	Moderate	17 (27.4)	13 (23.6)	
	High	3 (4.8)	0 (0.0)	

^1^ Chi-square test. Bold values indicate statistical significance (*p* < 0.05). Post hoc comparisons were performed using adjusted standardized residuals with Bonferroni correction. Different superscript letters within the same row indicate a statistically significant difference between groups (*p* < 0.05), whereas identical letters indicate no significant difference. Possible MCI, screening positive for possible mild cognitive impairment; MoCA, Montreal Cognitive Assessment; Q1–Q3, first to third quartiles.

**Table 3:** Anthropometric measurements and dietary characteristics according to the MoCA classification in older adults.

Variable	Possible MCI (*n* = 62)	Normal Cognitive Performance (*n* = 55)	Total (*n* = 117)	*p*-Value
Body fat percentage, %	39.98 (34.39–46.80)	38.20 (34.20–43.30)	39.50 (34.30–44.25)	0.093 ^1^
WC, cm	106.90 ± 10.98	105.06 ± 8.89	106.03 ± 10.05	0.323 ^2^
WHR	0.92 (0.88–1.00)	0.94 (0.90–1.00)	0.94 (0.89–1.00)	0.359 ^1^
BMI, kg/m^2^	32.67 ± 5.78	29.54 ± 3.39	31.20 ± 5.04	**<0.001** ^2^
DDS	6.00 (5.00–7.00)	6.00 (6.00–7.00)	6.00 (5.00–7.00)	0.910 ^1^
HEI-2020	47.75 ± 12.60	44.96 ± 12.03	46.44 ± 12.36	0.224 ^2^
Energy intake, kcal/day	1478.79 (1279.55–1959.80)	1695.08 (1422.45–2088.21)	1616.88 (1328.86–2012.13)	0.132 ^1^
Energy intake, kcal/kg/day	19.26 (14.85–25.46)	22.64 (18.87–28.80)	20.80 (15.77–26.70)	**0.029** ^1^
Protein intake, g/day	56.71 (43.60–77.77)	65.46 (50.49–81.40)	60.58 (46.13–80.49)	0.179 ^1^
Protein intake, g/kg/day	0.71 (0.50–1.06)	0.80 (0.67–1.03)	0.77 (0.58–1.04)	0.082 ^1^
Protein, % of energy	15.50 (13.00–18.00)	16.00 (13.00–18.00)	16.00 (13.00–18.00)	0.889 ^1^
BMI category				
Non-obese, *n* (%)	23 (37.1)	32 (58.2)	55 (47.0)	**0.027** ^3^
Obese, *n* (%)	39 (62.9)	23 (41.8)	62 (53.0)	

Bold values indicate statistical significance (*p* < 0.05). Data are presented as mean ± standard deviation for normally distributed variables and as median (Q1–Q3) for non-normally distributed variables, unless otherwise indicated. ^1^ Mann–Whitney U test; ^2^ independent-samples *t*-test; ^3^ Fisher’s exact test. BMI, body mass index; DDS, Dietary Diversity Score; HEI-2020, Healthy Eating Index-2020; possible MCI, screening positive for possible mild cognitive impairment; MoCA, Montreal Cognitive Assessment; Q1–Q3, first to third quartiles; WC, waist circumference; WHR, waist–hip ratio. BMI was categorized as non-obese (<30.0 kg/m^2^) and obese (≥30.0 kg/m^2^); the normal-weight and overweight categories were combined due to low cell frequencies.

**Table 4:** Spearman correlations of anthropometric and dietary variables with MoCA scores in older adults.

Variable	*r*_s_	*p*-Value
Body fat percentage, %	−0.162	0.080
WC, cm	−0.180	0.052
WHR	0.014	0.883
BMI, kg/m^2^	**−0.303**	**0.001**
DDS	0.058	0.536
HEI-2020	−0.057	0.539
Energy intake, kcal/day	0.117	0.210
Energy intake, kcal/kg/day	0.177	0.056
Protein intake, g/day	0.097	0.300
Protein intake, g/kg/day	0.127	0.172
Protein, % of energy	−0.040	0.670

Spearman’s rank correlation test. Bold values indicate statistical significance (*p* < 0.05). BMI, body mass index; DDS, Dietary Diversity Score; HEI-2020, Healthy Eating Index-2020; MoCA, Montreal Cognitive Assessment; r_s_, Spearman’s rank correlation coefficient; WC, waist circumference; WHR, waist–hip ratio.

**Table 5:** Hierarchical multiple linear regression analysis of sociodemographic, physical activity, anthropometric, and dietary factors associated with MoCA scores in older adults.

**(A) Model Summary**								
**Model**	**R^2^**	**Adjusted R^2^**	**ΔR^2^**	**ΔF**	***p*** **for ΔF**	**Model F**		**Model *p***
Model 1	0.197	0.168	0.197	6.864	<0.001	F(4, 112) = 6.864		<0.001
Model 2	0.238	0.204	0.042	6.060	0.015	F(5, 111) = 6.952		<0.001
Model 3	0.260	0.212	0.021	1.558	0.215	F(7, 109) = 5.460		<0.001
**(B) Coefficients of the Final Model**								
**Variable**	**B**	**SE**	**β**	**t**	***p*** **-Value**	**95% CI for B**	**Tolerance**	**VIF**
Constant	22.976	9.795	–	2.346	0.021	3.562 to 42.390	–	–
Age, years	−0.012	0.109	−0.009	−0.106	0.916	−0.227 to 0.204	0.953	1.050
Sex	−0.694	1.244	−0.051	−0.558	0.578	−3.160 to 1.772	0.816	1.226
Years of education	0.768	0.180	0.363	4.277	**<0.001**	0.412 to 1.124	0.945	1.058
Physical activity, MET-min/week	−0.001	0.000	−0.161	−1.859	0.066	−0.002 to 0.000	0.907	1.103
BMI, kg/m^2^	−0.301	0.114	−0.240	−2.632	**0.010**	−0.527 to −0.074	0.819	1.221
DDS	0.673	0.480	0.124	1.403	0.163	−0.278 to 1.624	0.865	1.155
HEI-2020	−0.068	0.045	−0.132	−1.492	0.139	−0.157 to 0.022	0.866	1.155

Bold values indicate statistical significance (*p* < 0.05). Dependent variable: MoCA score. Model 1 included age, sex, years of education, and physical activity. Model 2 additionally included BMI; Model 3 additionally included DDS and HEI-2020. For Model 1, ΔR^2^ and ΔF refer to changes relative to the intercept-only model. Sex was coded as male = 1 and female = 2. Durbin–Watson statistic = 1.689. B, unstandardized coefficient; β, standardized coefficient; BMI, body mass index; CI, confidence interval; DDS, Dietary Diversity Score; HEI-2020, Healthy Eating Index-2020; MoCA, Montreal Cognitive Assessment; SE, standard error; VIF, variance inflation factor.

**Table 6:** Sensitivity analyses assessing the robustness of the association between anthropometric indicators and cognitive performance following adjustment for dietary factors and comorbidities.

**(A) Anthropometric Indicator (Block 2)**								
**Model**	**Indicator**	**B (95% CI)**			**β**	***p*** **-Value**	**ΔR^2^**	***p*** **for ΔR^2^**
BMI model	BMI	−0.281 (−0.507 to −0.055)			−0.224	**0.015**	0.042	**0.015**
WC model	WC	−0.052 (−0.161 to 0.057)			−0.083	0.342	0.007	0.342
WHR model	WHR	−3.556 (−18.581 to 11.469)			−0.046	0.640	0.002	0.640
**(B) Dietary Block (Block 3) and Final Model Fit**								
**Model**	**DDS β**	***p*** **-Value**	**HEI-2020 β**	***p*** **-Value**	**ΔR^2^**	***p*** **for ΔR^2^**	**Final R^2^**	**Adjusted R^2^**
BMI model	0.124	0.163	−0.132	0.139	0.021	0.215	0.260	0.212
WC model	0.109	0.234	−0.106	0.244	0.015	0.356	0.218	0.168
WHR model	0.109	0.234	−0.108	0.238	0.015	0.351	0.214	0.163
**(C) Additional Adjustment for Comorbidities**								
**Model**			**BMI B (95% CI)**		**β**	***p*** **-Value**	**Final R^2^**	**Adjusted R^2^**
Primary model			−0.301 (−0.527 to −0.074)		−0.240	0.010	0.260	0.212
Comorbidity-adjusted model			−0.305 (−0.537 to −0.072)		−0.243	**0.011**	0.272	0.196

Bold values indicate statistical significance (*p* < 0.05). Dependent variable: MoCA score. In Panels A and B, all models included age, sex, years of education, and physical activity in Block 1; the specified anthropometric indicator in Block 2; and DDS and HEI-2020 in Block 3. The β and *p* in Panel A are from Block 2, whereas those in Panel B are from the final model. Panel C compares the final BMI model with the corresponding model additionally adjusted for hypertension, diabetes mellitus, hypercholesterolemia, and cardiovascular disease. In this model, in addition to age, sex, years of education, physical activity, DDS, and HEI-2020, further adjustments were made for diabetes mellitus and hypertension, as well as hypercholesterolemia and cardiovascular disease. Final model statistics for the BMI, WC, and WHR models were F(7, 109) = 5.460, F(7, 109) = 4.351, and F(7, 109) = 4.233, respectively (all *p* < 0.001). The corresponding Durbin–Watson statistics were 1.689, 1.531, and 1.496. For the comorbidity-adjusted model in Panel C, the final model statistic was F(11, 105) = 3.573, *p* < 0.001, with a Durbin–Watson statistic of 1.670. BMI, body mass index; CI, confidence interval; DDS, Dietary Diversity Score; HEI-2020, Healthy Eating Index-2020; MoCA, Montreal Cognitive Assessment; WC, waist circumference; WHR, waist–hip ratio.

## Data Availability

The data supporting the findings of this study are available from the authors upon reasonable request. Owing to privacy restrictions, the data are not publicly available. Interested researchers can contact the authors to discuss the terms of access and usage.

## Abbreviations

The following abbreviations are used in this manuscript:

APOE	apolipoprotein E
BMI	body mass index
CI	confidence interval
DDS	Dietary Diversity Score
FAO	Food and Agriculture Organization of the United Nations
FINGER	Finnish Geriatric Intervention Study to Prevent Cognitive Impairment and Disability
HEI-2020	Healthy Eating Index-2020
IPAQ-SF	International Physical Activity Questionnaire–Short Form
MCI	mild cognitive impairment
MET	metabolic equivalent of task
MIND	Mediterranean–DASH Intervention for Neurodegenerative Delay
MoCA	Montreal Cognitive Assessment
Q1–Q3	first to third quartiles
SE	standard error
STROBE	Strengthening the Reporting of Observational Studies in Epidemiology
TPAS	total physical activity score
VIF	variance inflation factor
WC	waist circumference
WHR	waist–hip ratio
